# Contamination of *Zophobas morio* Larvae Rearing Substrate with *Listeria monocytogenes*: A Preliminary Study

**DOI:** 10.3390/ani13071198

**Published:** 2023-03-29

**Authors:** Filippo Fratini, Luca Ciurli, Mario Forzan, Ata Kaboudari, Emma Copelotti, Gisella Paci, Simone Mancini

**Affiliations:** 1 Department of Veterinary Sciences, University of Pisa, Viale delle Piagge 2, 56124 Pisa, Italymario.forzan@unipi.it (M.F.); a.kaboudari@urmia.ac.ir (A.K.); emma.copelotti@phd.unipi.it (E.C.); gisella.paci@unipi.it (G.P.); simone.mancini@unipi.it (S.M.); 2 Interdepartmental Research Center “Nutraceuticals and Food for Health”, University of Pisa, Via del Borghetto 80, 56124 Pisa, Italy; 3 Department of Food Hygiene and Quality Control, Urmia University, Urmia P.O. Box 1177, Iran

**Keywords:** edible insects, *Listeria monocytogenes*, superworm, novel food, food safety

## Abstract

**Simple Summary:**

Over the past 20 years, we have seen exponential growth in the number of scientific publications on edible insects as an alternative food source. Usually, insect larvae are reared in a close environment in constant contact with their feed, called the substrate. The substrates employed for rearing could represent one of the most important ways to involuntarily introduce risk in the production chain. In this research, we inoculated one of the most important food pathogens, *Listeria monocytogenes*, in the substrates used in *Zophobas morio* rearing (also called superworm). The results highlighted the caution needed in insects rearing and the potential risk related to this pathogen in relation to superworm rearing. Attention was also focused on the technological procedure employed in insect rearing and processing.

**Abstract:**

The interest in edible insects is continuously increasing due to their environmental, nutritional, and productive features. The aim of this study was to evaluate the effects and survival of *Listeria monocytogenes* in *Zophobas morio* rearing, using two different bacterial loads (2 and 6 log CFU/g). We also considered the effect of washing, fasting, and cooking treatments on the larvae. During the experimental trial, no mortality was observed among the larvae. The *L. monocytogenes* loads decreased over time, and it was below the detection limit for crates inoculated with 2 log CFU/g, while the substrate inoculated with 6 log CFU/g reached loads of 4.26 (without larvae) and 2.83 log CFU/g (with larvae). Due to the absence of *L. monocytogenes* in the larvae on day seven, it was not possible to assess the fasting treatment or the washing and the cooking. However, when looking at the total microbial count, significant effects were revealed for all treatments. The unfasted larvae showed no effect of washing, while the total microbial counts decreased after washing in the fasted larvae. This proves that fasting is a good treatment in terms of hygiene assurance for the consumer.

## 1. Introduction

Today, the livestock production is one of the most employable sectors in the world: it supports 600 million smallholder farmers in developing countries; it relies on 1.3 billion of workers globally; and provides 17 percent and 33 percent of kilocalories and protein consumption worldwide, respectively [1]. It has a total economic value of about USD 1.4 trillion. Due to the population growth and urbanization, in the last 40 years, the global meat production has been increasing at an annual rate of about 5 percent, especially among developing regions, causing dramatic changes in the environment: 30 percent of the ice-free terrestrial surface has now been taken up to produce meat, which has tripled its amount from 45 to 134 million tons between 1980 and 2002 [2].

The main environmental concerns related to livestock are the (i) eutrophication as a common consequence of meat production due to the impossibility of naturally eliminating the manure; (ii) land use: although arable land is able to produce a sufficient amount of vegetables for humans consumption, it is not self-sufficient for animal purposes, reaching only 20% protein production; (iii) the energy consumed to produce and transport food and to keep operative animal facilities, slaughterhouses, etc.; (iv) water consumption as animal farming and agriculture account for 70% of the worldwide water used, whereas industry and domestic uses account for 22 and 8% respectively [3,4,5]. Concerning greenhouse emissions, animal, feed, and agriculture production contribute to the total methane and nitrous oxide gases in the atmosphere. 

In the last 20 years, there has been exponential growth in the number of publications concerning edible insects as an alternative food source, describing their ecological, economic and nutritional opportunities [6]. Considering greenhouse emissions, energy consumption, and land use, insects, such as *Tenebrio molitor* (mealworms), do not produce methane at all and have a lower global warming potential (GWP) per kilogram of edible protein than milk (1.77–2.80 times the value for mealworm), chicken (1.32–2.67), pork (1.51–3.87), and beef (5.52–12.51). Although the energy used for insects rearing is lower than that for beef (1.02–1.58 times the value for mealworm), it is higher than that for milk (46–88% of the mealworm value) and chicken (46–88%), and it is similar to that of pork (55–137%). Energy use deeply depends on the insect species, physical needs, and geographical region; indeed, temperature affects insect metabolism and development, so a large part of the energy is used to ensure the correct farming environment. The land used for insect rearing much smaller compared with that for milk (1.81–3.23 times the value for mealworms), chicken (2.30–2.85), pork (2.57–3.49), and beef (7.89–14.12).

Finding an alternative source of protein to meat is really important in terms of ecology due to excessive consumption: the daily European mean intake is above the population reference intake of 0.83 g/kg body mass. In 1992, the United Nation published the Agenda 21; during the Conference on Environment and Development, the UN showed how patterns of energy, environmental damage, poor health, poverty, and underdevelopment are strictly linked together and can lead to a deadly knot. All these themes have brought certain countries into a deep food insecurity crisis, which means that a high percentage of the population may starve for a day or more without proper government strategy. Both food security (daily access to the needed food) and food safety (food nutritional characteristics and whether it endangers health owing to biological, chemical, or physical hazards) play a key role in food insecurity.

*Listeria monocytogenes* is the causative agent of listeriosis, a dangerous foodborne disease of important health concern for humans [7]. Listeriosis is mainly a foodborne disease both in humans and animals, and the bacteria can be isolated even in healthy subjects. Many different food sources can be a vector for these bacteria, for example, meat, cheese, fish and shellfish, and vegetables [7]. According to the Centers for Disease Control and Prevention (CDC), in 1999 in the U.S., *L. monocytogenes* was the main cause of hospitalization, among foodborne pathogens, accounting for 90.5 percent and a fatality rate of 21 percent. It is the primary pathogen responsible for listeriosis infection, accounting for 99 percent of the total [8]. Data from the EFSA confirmed that this bacterium is still current a risk: in 2021, listeriosis was the fifth most reported zoonosis in the EU, with 2183 cases (a 14% increase in the EU compared with 2020), 923 hospitalizations, and 196 deaths [9].

The host ingests contaminated food, and this passes through the stomach; then, *L. monocytogenes* replicates in the intestine and settles in the liver [10]. In healthy people, it mainly causes gastroenteritis [11]. Notably, promising findings in the detection of pathogenic bacteria in edible insects have been reported. *Salmonella* spp., *Listeria* spp. and *Bacillus cereus* were never found in reared or processed insects; only a few researchers found coagulase-positive staphylococci in insects reared for feed/food in a controlled setting (laboratory-scale farming or food industries) [12,13,14]. Notably, Vandeweyer et al. [15] reported that it is important to estimate the risks for coincidental contamination during insect rearing or processing by a certain pathogen.

Two studies tested the persistence of *Listeria* spp. in mealworms after spiking the substrates with live bacteria cells. Mancini et al. [16] reported that *L. monocytogenes* could persist during the rearing period. The authors reported that the load of this bacteria could be reduced via a fasting period of 24–48 h, while washing the larvae was ineffective. Moreover, cooking the larvae in an oven for 10 min at 150 °C reduced the *L. monocytogenes* load to under the detection limit, reducing the risk associated with this foodborne pathogen to zero. Similarly, Belleggia et al. [17] reported that *L. innocua* could establish in the rearing environment and multiply in both the rearing substrate and larvae gut. As previously reported, washing the larvae was ineffective, while heat treatments such as boiling, oven cooking, and deep frying were effective in the reduction of the bacterial load. The capability to resist *L. monocytogenes* infection by the larvae, and therefore their capacity to indirectly become a vector of the pathogen, was also demonstrated by Edosa et al. [18], who tested the capacity of the larvae to express an autophagy-related gene (*TmAtg6*) that plays an essential role in antimicrobial defense against intracellular bacterial infection.

*Zophobas morio* (superworms) larvae could be also an interesting edible insect as they are very similar to mealworms in terms of rearing requirements and nutritional value. This insect, as mealworms, belongs to the Tenebrionidae family, and its larvae show an excellent chemical composition, with a high content of proteins, about 45% of dry matter (DM), and an equal content of lipids, about 43% of DM [19,20,21]. Superworms have several features similar to those of mealworms, and in the rearing and technological characteristics, such as the plasticity to adapt to different substrates and the susceptibility to blanching treatments [20,21,22].

One of the future goals of the insect sector is to employ former foodstuffs containing meat and fish as substrates. These substrates could, on one hand, contribute to the environmental impact of the sector, leading to reducing the waste material and closing the production chain [23]. On the other hand, these materials could represent a vehicle of microorganisms, such as *Listeria* and other pathogens, that currently are not detected in insect products [24].

Therefore, considering the present rising interest in insects as a source of food and feed, the aim of this study was to assess the persistence and behavior of *L. monocytogenes* in the rearing substrate of *Z. morio* larvae. Technological treatment effects, such as for fasting, washing, and cooking, were also analyzed.

## 2. Materials and Methods

### 2.1. Bacterial Strain and Microbial Quantifications

*L. monocytogenes* ATCC 7644 strain was used. The strain was stored at −80 °C in brain heart infusion (BHI) broth (Thermo Fisher Scientific, Milan, Italy) in a 10% glycerol suspension. The strain was cultured in BHI broth for 24 h at 37 °C in aerobic conditions. Following the method reported by Mancini et al. [16], several tubes were cultured in order to obtain cellular pellets that were gathered together, reaching a final *L. monocytogenes* load about of 8 log CFU/mL. Enumeration of *L. monocytogenes* was carried out by spreading 0.1 mL of the ten-fold serial dilutions on Agar Listeria Ottaviani Agosti (ALOA) plates (Biolife Italiana Srl, Milan, Italy). The plates were incubated at 37 °C for 48 h, and the results are expressed as log CFU per gram or milliliter. The total microbial load was evaluated by sowing progressive dilutions of the samples on agar plate count (APC) plates (Oxoid, Milan, Italy); plates were then incubated for 72 h at 30 °C.

### 2.2. Experimental Design

*Z. morio* larvae were reared at the Department of Veterinary Science, University of Pisa, in plastic crates of 39 × 28 × 14 cm. The farming temperature was set to 25 °C (±1) with a relative humidity range of 55–65%. The larvae were fed a mix of spent brewery grains and bread leftover (1:1; dry matter, DM: 97.08%; ether extract: 2.26% of DM; crude protein: 14.20% of DM; ash: 2.47% of DM).

**Figure 1:**
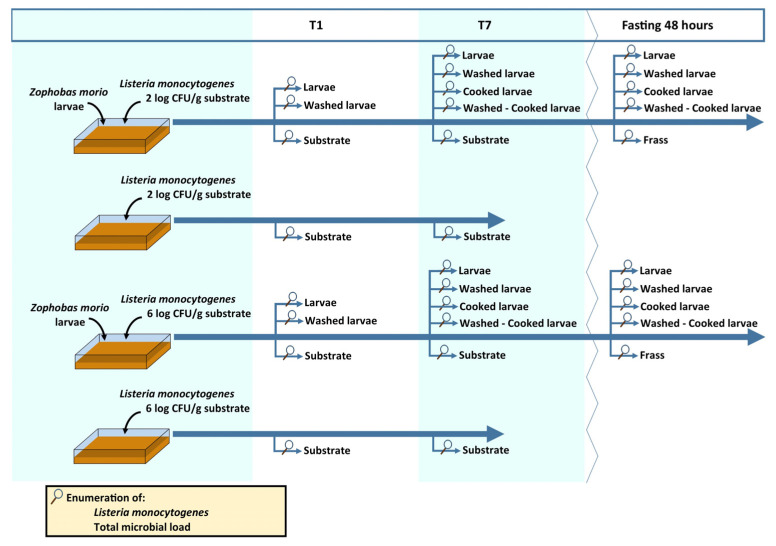
Experimental design of the trial. T1: sampling after 1 day; T7: sampling after 7 days; fasting 48 h: sampling of fasted larvae derived from T7.

Two bacterial inocula were employed in the trial, reaching final loads of 2 and 6 log CFU/g of substrate. The bacterial cell solutions were poured onto the rearing substrate (50 g), and then the mix was homogenated before larvae addition. Four series of crates were made, two per inoculum, with and without larvae addition, as reported in Figure 1. The required 100 g of larvae (approximately 100 larvae) were added into the crates. All the combination inoculum–larvae were replicated 3 times, for a total of 12 crates. Microbiological determinations were carried out on the larvae and rearing substrates after 1 and 7 days (T1, T7) starting from *L. monocytogenes* contamination. Larvae and substrate were tested prior to the beginning of the trial for the absence of *Listeria* spp. and total bacterial load (about 10^8^ CFU/g for the larvae, while the substrates showed total bacterial load below the detection limits due to the employment of ingredients that were previously oven dried). The larvae were fasted, washed, and cooked as reported below; all the interactions between inocula (*L. monocytogenes* at 2 and 6 log CFU/g of substrate), fasting (yes/no), washing (yes/no), and cooking (yes/no) were tested (Figure 1). The larvae were fasted for 48 h in sterile plastic containers with a plastic web as a base. Frass was collected in a second sterile plastic container placed below the plastic web. Washing was performed in sterile stomacher bags with a ratio larvae to sterile saline solution of 1:9. The bag was thoroughly shaken for 3 min, and then the washing solution was removed by pipetting. The washed larvae were collected and used for microbial determination. Larvae were cooked in a preheated oven at 150 °C for 10 min.

### 2.3. Statistical Analyses

Two-was ANOVA was performed to assess the effect of the inoculum (*L. monocytogenes* at 2 and 6 log CFU/g of substrate), time (T1 and T7), and larvae presence (with or without) on the substrate loads. The effects of the inoculum, time, washing, and cooking were tested by ANOVA taking into consideration all the interactions between the factors. Moreover, in order to highlight the single factor effect, Student’s *t* tests were performed. Statistical significance was set at 0.05, and differences were assessed using Tukey’s test. R free statistical software was used [25].

## 3. Results and Discussion

**Figure 2:**
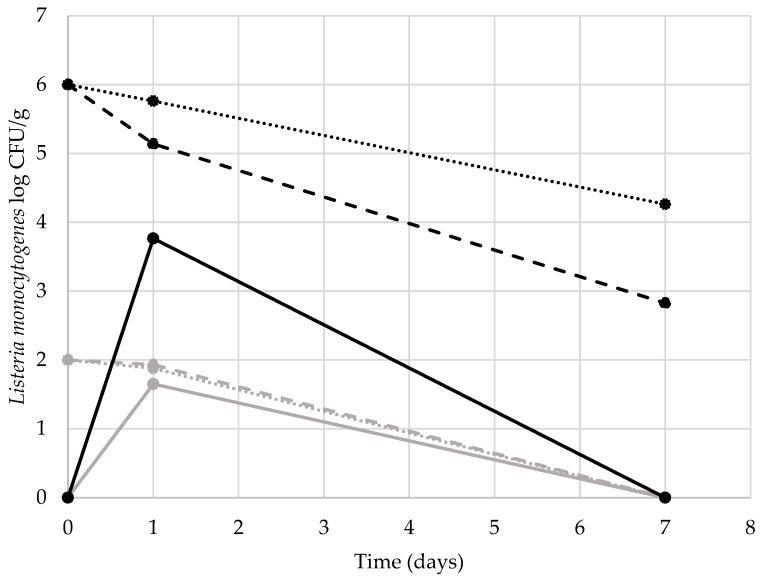
*Listeria monocytogenes* counts in the substrates (with and without larvae) and larvae. *Listeria monocytogenes* inocula of 2 log CFU/g and 6 log CFU/g, respectively, in grey and black. The dotted lines represent the counts in the substrate without the larvae, the dashed lines represent the counts in the substrate with the larvae, and the full lines represent the counts of the larvae.

**Figure 3:**
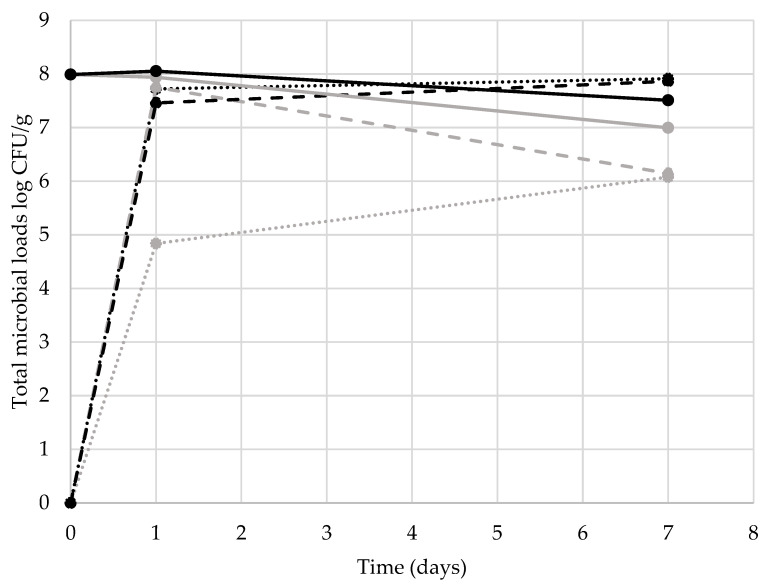
Total microbial loads in the substrates (with and without larvae) and larvae. *Listeria monocytogenes* inocula of 2 log CFU/g and 6 log CFU/g, respectively, in grey and black. The dotted lines represent the counts in the substrate without the larvae, the dashed lines represent the counts in the substrate with the larvae, and the full lines represent the counts of the larvae.

During the trial, no mortality occurred among the larvae contaminated with either inoculum, suggesting that *L. monocytogenes* did not affect *Z. morio* viability for this concentration, or at least for 6 log CFU/g or less. Similar results were reported in the larvae of *Tenebrio molitor* reared in substrates inoculated with *L. monocytogenes*, *L. innocua*, or *Salmonella* spp. [16,17,26]. The results of the quantifications of *Listeria monocytogenes* and the microbial load are reported in Figure 2 and Figure 3, respectively.

For *L. monocytogenes,* statistically significant differences were detected between the loads of the inocula both on the substrates and on the larvae, as well as between the tested days. *L. monocytogenes* load decreased in the substrates without larvae with a log CFU/g at T1 of 5.76 and 1.88, respectively, for the inocula of 6 and 2 log CFU/g. Similarly, the loads decreased in the substrate with the larvae reaching 5.14 and 1.88 log CFU/g, respectively. After seven days, the *L. monocytogenes* load was under the detection limit for the substrates spoiled with 2 log CFU/g (with and without larvae), while the substrate inoculated with the higher concentration of the bacteria reached loads of 4.26 when the larvae were absent and 2.83 when the insects were fed the substrate. At T1, the larvae showed a load of *L. monocytogenes* of 1.65 and 3.77 log CFU/g, respectively, for the inoculated substrate of 2 and 6 log CFU/g. At both *inocula* levels, the larvae did not carry the bacteria at T7. These results are in contrast with those of Belleggia et al. [17] and Mancini et al. [16] in mealworms, who highlighted the persistence of *L. innocua* (at 1, 5, and 7 log CFU/g) and *L. monocytogenes* (at 8 log CFU/g) for 7 days. Therefore, some differences in the two insect species must produce different responses to the presence of the bacteria species. The level of contamination and the trial length could also have affected the bacterial dynamics, as also reported by Wynants et al. [26] in mealworm larvae reared in substrates spoiled with a mix of *Salmonella enterica* serovar Enteritidis (LMG 18,735), *Salmonella enterica* serovar Typhimurium (LMG 18,732), and *Salmonella enterica* serovar Infantis (LMG 18,746).

**Figure 4:**
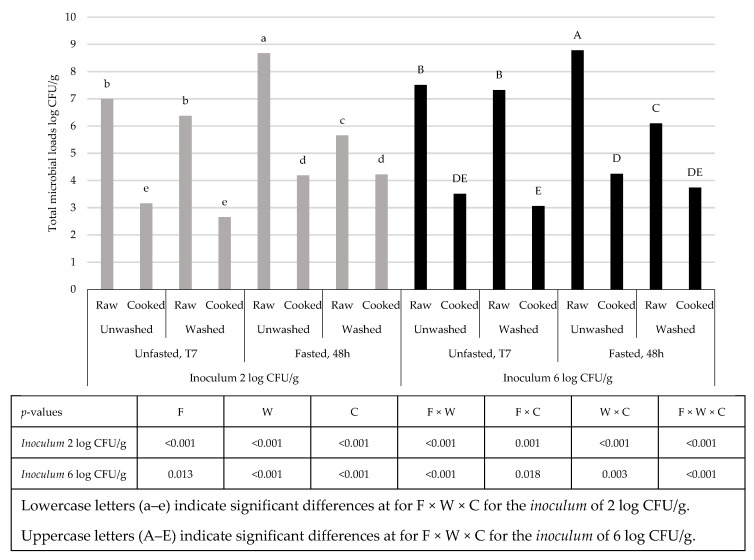
Effects of fasting (F), washing (W), and cooking (C) on the total microbial loads of the larvae reared in the substrates inoculated with *Listeria monocytogenes*.

The total microbial counts at T1 of the substrate without the larvae were 4.84 and 7.72 log CFU/g for the *inocula* 2 and 6 log CFU/g of *L. monocytogenes,* respectively. The substrates with the larvae showed total counts of 7.75 log CFU/g (2 log inocula) and 7.46 log CFU/g (6 log inocula). At T7 the substrates showed total loads of 6.08 and 7.91 log CFU/g for the crates without the larvae and of 6.15 and 7.87 log CFU/g for the crates with the larvae. *Z. morio* larvae reared in the substrates inoculated with *L. monocytogenes* reached loads of 7.94 and 8.05 log CFU/g at T1 and 7.00 and 7.51 log CFU/g at T7 (for the inocula of 2 and 6 log CFU/g of *L. monocytogenes,* respectively). No effect of washing was highlighted at T1, as the washed larvae presented similar *L. monocytogenes* and total microbial counts to the unwashed ones (1.67–3.77 and 8.04–7.91 log CFU/g, respectively, for the inoculated substrate of 2 and 6 log CFU/g for the counts of *L. monocytogenes* and total microbial load). The lack of effect of washing in reducing the *Listeria* spp. loads in Tenebrionidae larvae is in accordance with the results of Mancini et al. [16] on *L. monocytogenes* and of Belleggia et al. [17] on *L. innocua* in mealworm larvae. These results suggest that *Listeria* cells are harbored in the larval gut rather than the external cuticle. Similarly, Wynants et al. [27] reported a lack of effect of rinsing on microbial numbers in mealworm larvae. *L. monocytogenes* cells were found in the larvae frass after starvation for 48 h in both the level of contamination at 3.95 and 4.97 log CFU per gram of frass (for larvae derived from the rearing substrate contaminated at 2 and 6 log CFU/g, respectively). These data are in line with those on mealworms reared in inoculated substrate [16,17]. Similarly, the total microbial loads of frass were in line with those previously reported by Mattioli et al. [28] (8.82 and 9.22 log CFU per gram of frass, respectively, for the two levels). Due to the absence of *L. monocytogenes* in all the larvae samples at T7, it was not possible to evaluate the effect of the fasting treatment as well as the effect of washing and cooking on this pathogen. However, looking at the total microbial counts, significant effects of fasting, washing, and cooking were highlighted (Figure 4).

Unfasted larvae showed no effect of washing, while the total microbial loads were reduced after washing of fasted larvae. These differences could be ascribed to the fact that, during fasting, larvae continue moving inside crates, inducing a positive “grooming” that allowed the reduction in the external pollution deposited on the larvae surfaces that was removed by the washing solution. Indeed, the effect of fasting alone did not induce a reduction in total viable counts; on the contrary, it probably led to a reduction in the ratio body mass:gut, as the log CFU per gram increased after 48 h of starving. Cooking decreased the counts in all the larvae without differences compared with the previous treatments, reaching levels ranging between 2.66 and 4.25 log CFU/g, with an average decrease of 3.57 log CFU/g. The effects of the different treatments are in line with those of previous researchers and with the recommendation from the EFSA to proceed with a fasting step in edible insects before their use as food [16,26,27,29,30,31,32,33].

## 4. Conclusions

In conclusion, *L. monocytogenes* appears to have no negative effect on the viability of *Z. morio* larvae. This result is promising in terms of the management of potential breeding, even on a large scale, where the probability of contamination is higher. It was not possible to evaluate the effect of the cooking or fasting treatments on *L. monocytogenes* due to their absence in both tests at T7 and after the fasting treatment, although the results of the latter on the total bacterial load led to significant decreases, again indicating its value as an important sanitizing treatments for edible insect larvae. The effect of the reported washing treatment, on the other hand, did not produce statistically significant results; therefore, it appears to be of little value in the procedures, which is noteworthy from the point of view of the sustainability of a farm by allowing reduced water use. On the basis of these data, which are preliminary, *Z. morio* appears to be an optimal candidate among the various edible insects entering the European market with regard to potential contamination by *L. monocytogenes* should this occur along the production chain. In the future, tests could be carried out to assess the presence of inhibitory molecules such as antimicrobial peptides (AMPs) that may be responsible for this strong decrease in the pathogen even from very high concentrations in the substrate.

## Data Availability

The raw data are available from the corresponding author upon reasonable request.
